# Account of the Operation for Aneurism of the Subclavian Artery, Performed by Mr. Brodie

**Published:** 1823-04

**Authors:** 


ART. IX.—*Account of the Operation for Aneurism of the Subclavian Artery, performed by* Mr. BRODIE. By the EDITORS.

WE had the pleasure of witnessing the operation of tying the subclavian artery, by Mr. Brodie, at St. George's Hospital, on the 7th of this month, (March.) There were some very interesting points in this operation, which was performed, in a very masterly style, in the space of sixteen minutes. The patient was a man about sixty, to all appearance healthy; the aneurism was not of large size, and the artery at the wrist of the affected limb, pulsated effectively, but not quite so strongly as in the other arm. The first incision was made in the usual direction, and *nearly* in a straight line, which appears to give greater facility than one more curved: the skin had previously been drawn downwards, by which the risk of wounding the external jugular was avoided. Bleeding from the numerous venous branches had always hitherto been found to obscure and retard the performance of this operation; and therefore Mr. Brodie applied a double ligature upon the external jugular vein, which vessel he afterwards divided in the interval between the two; by which means the succeeding steps of the operation were rendered comparatively easy. The ligature was placed under the artery, by means of Mr. Weiss's newly-invented aneurismal needle, which here, as well as in the case lately operated upon by Mr. Travers, completely answered the expectations of the surgeon, and of the ingenuity and simplicity of which we must beg to express our most unqualified approbation. On tightening the ligature, the pulsation both in the tumor and at the wrist were immediately stopt.

We have learnt with regret that the subject of this skilful operation died on the night between the 12th and 13th, although the symptoms were so favourable at first as to lead to the expectation of a different result. We understand that, on dissection, a considerable coagulum was found in the artery, extending some way on either side of the ligature, which was separating favourably: there was neither venal nor arterial inflammation, nor any diseased appearance of the nerves. The surface of the wound was sloughy; the opposite arm and fore-arm (that is, the right, the operation having been performed on the left,) were in a state of gangrene; and, on cutting into the muscles of the limb, they were found loaded with blood, as if they had been bruised: there was also a gangrenous appearance of the left arm, near the inner condyle of the humerus, but only to a small extent.

We forbear to enter into further particulars, not doubting but that Mr. Brodie himself will detail this very interesting case more fully. We have had the satisfaction to learn that an old friend and associate, Mr. (now Dr.) Gibbes, of St. Petersburgh, has lately performed this operation with success.

